# Menstrual Headache in Women with Chronic Migraine Treated with Erenumab: An Observational Case Series

**DOI:** 10.3390/brainsci11030370

**Published:** 2021-03-13

**Authors:** Raffaele Ornello, Ilaria Frattale, Valeria Caponnetto, Eleonora De Matteis, Francesca Pistoia, Simona Sacco

**Affiliations:** 1 Neuroscience Section, Department of Applied Clinical Sciences and Biotechnology, University of L’Aquila, 67100 L’Aquila, Italy; raffaele.ornello@gmail.com (R.O.); valeria.caponnetto@univaq.it (V.C.); eleonora.dematteis@graduate.univaq.it (E.D.M.); francesca.pistoia@univaq.it (F.P.); 2 Child Neurology and Psychiatry Unit, Systems Medicine Department, Tor Vergata University, 00133 Rome, Italy; ilariafrattale@libero.it

**Keywords:** migraine, calcitonin gene-related peptide, chronic migraine, menstrual migraine, monoclonal antibodies

## Abstract

Background: We aimed to assess the differences between menstrual and non-menstrual headache in women with chronic migraine treated with erenumab. Methods: We included fertile women from a single center. Patients were defined as responders to erenumab if reporting a ≥50% decrease in monthly headache days, as compared to pre-treatment for more than half of the treatment period. Premenstrual days were defined as the two days preceding menstruation, while menstrual days were defined as the first three days of menstruation. Results: We included 18 women (11 erenumab responders and 7 erenumab non-responders) contributing to a total of 103 menstrual cycles and 2926 days. The proportion of headache days was higher in menstrual than in premenstrual and non-menstrual days in erenumab responders (34.4% vs. 14.8% vs. 16.3%, respectively; *p* < 0.001) and in erenumab non-responders (71.4% vs. 53.6% vs. 48.3%, respectively; *p* < 0.001). Headache days with ≥2 acute medications were higher in menstrual than in premenstrual or non-menstrual headache days in erenumab non-responders (*p* = 0.002) but not in erenumab responders (*p* = 0.620). Conclusions: Our data suggest that migraine is more frequent during than outside menstrual days even in women treated with erenumab.

## 1. Introduction

Migraine is a headache disorder affecting 14% of people worldwide [1]. This disorder can manifest itself with variable degrees of frequency and severity. The most severe form is chronic migraine, in which headache occurs on ≥15 days each month [2]. Migraine is often associated with vascular [3,4], psychiatric [5,6], and gastrointestinal comorbidities [7] which might, in turn, have a negative impact on the course of headache. Migraine prevalence and related disability is particularly high in young women due to the presence of high estrogen levels and their frequent fluctuations [8]. Migraine occurs more frequently during the days around the menstruation than in the other periods of the menstrual cycles in fertile women [9,10], due to a sudden fall in estrogen levels—the so-called “estrogen withdrawal” [11]. Compared with non-menstrual migraine episodes, menstrual episodes are longer, more severe, and more refractory to acute treatments [12]. The menstrual pattern of migraine is so typical that the International Classification of Headache Disorders includes the definitions of “pure menstrual migraine” and “menstrually-related migraine” to identify migraines that occur only or preferentially during the days around the menstruation [2].

Calcitonin gene-related peptide (CGRP), which is released after the activation of the trigeminovascular system, is one of the key mediators of migraine pain [13]. Monoclonal antibodies antagonizing CGRP or its receptor are the first approved migraine-specific preventive agents [14,15]. Assessing the effect of those drugs on menstrual migraine attacks is important to determine the need for additional preventive measures, such as hormonal treatments.

To determine whether the difference between menstrual and non-menstrual migraine persists under treatment with a migraine-specific preventive agent, we performed a within-woman analysis in patients treated with erenumab, a monoclonal antibody antagonizing the CGRP receptor.

## 2. Materials and Methods

### 2.1. Study Population

The present study is a within-woman analysis from a real-life observational study on the efficacy and safety of erenumab [16,17]. The study was approved by the Internal Review Board of the University of L’Aquila; Reference number 44/2019. Each patient signed an informed consent. All anonymized data are available upon reasonable request to the corresponding author.

We included women aged ≥18 years, who were referred to our tertiary Headache Center from January 2019 to June 2020 with a diagnosis of chronic migraine, reporting regular menstrual cycles, and reporting menstruation as a trigger of more severe headache attacks. Women were treated with erenumab according to clinical indication [14]. To be included in the study, all women had to be free from medical comorbidities. To only assess the effect of natural menstrual cycles, without the influence of exogenous hormones [18], we excluded women who were taking oral contraceptives during the study period. We also excluded women in menopause. Erenumab samples were dispensed from the manufacturing company and were free of charge to patients. Patients started erenumab at the dose of 70 mg monthly; escalation to 140 mg monthly was allowed according to clinical needs. As this was an observational study, no treatment was mandatory, and concurrent oral preventive treatments were allowed. Women were defined “responders” to erenumab if reporting a ≥50% decrease in monthly headache days, as compared to the reported baseline for more than half of the treatment follow-up.

### 2.2. Data Collection

We collected the daily diaries in which patients were asked to annotate headache days, pain intensity according to a 0–10 Numerical Rating Scale, the number of acute medications used, and days of menstruation. We defined “moderate-to-severe” headache days as those days with a ≥4 score on the Numerical Rating Scale. We did not ask women to distinguish migraine from other headaches.

We included in the study women who reported days of menstruation for at least three cycles. Diary data were entered into a computerized database by a single operator.

According to the definition of the International Classification of Headache Disorders [2], we considered the five days around the first day of menstruation—i.e., days −2 to +3—to assess perimenstrual days. We defined the two days preceding the menstruation as “premenstrual” and the first three menstruation days as “menstrual”.

### 2.3. Statistical Analysis

We considered the observation day as the statistical unit. We recorded the number of headache days (including migraine and non-migraine), the number of acute medications per each headache day, and headache intensity. Those parameters were reported for menstrual, perimenstrual, and non-menstrual days. We calculated the odds ratios (ORs) and 95% confidence intervals of menstrual and premenstrual vs. non-menstrual headache days and moderate-to-severe headache days. We also calculated the ORs and 95% confidence intervals for days with ≥2 acute medication use, over the total days of observation. Proportions of headache days, moderate-to-severe headache days, and days with ≥2 acute medication use were compared using the Chi-square test. *p*-values were set at <0.05 for significance. The analyses were performed using SPSS version 20.0 (IBM, Armonk, NY, USA).

## 3. Results

We included in the study 18 women, with a median age of 38 years (interquartile range, 28–47 years), a median migraine duration of 18 years (interquartile range, 12–27 years), and a median of 19 years (interquartile range, 15–25 years) monthly headache days, before starting the erenumab treatment. The included women contributed a total of 2926 days and 103 menstrual cycles, with a mean (±standard deviation) cycle length of 27.3 ± 6.5 days; 309 days were considered menstrual, 206 premenstrual, and 2411 non-menstrual. Eleven of the 18 women were classified as erenumab responders and seven as erenumab non-responders; erenumab responders had 183 menstrual, 122 premenstrual, and 1414 non-menstrual days, while erenumab non-responders had 126 menstrual, 84 premenstrual, and 997 non-menstrual days. Table 1 reports the patients’ characteristics. The characteristics of erenumab responders did not differ from those of erenumab non-responders, except from a lower proportion of patients treated with the 140 mg monthly dose (*p* = 0.013). Three women escalated the dose from 70 mg to 140 mg monthly at the second administration, two at the third, two at the fourth, and four at the fifth administration. Medication overuse withdrawal occurred in all five erenumab responders and in none of the six erenumab non-responders reporting previous medication overuse. Notably, non-responders used concomitant oral preventatives more frequently than responders (Table 1).

**Figure 1:**
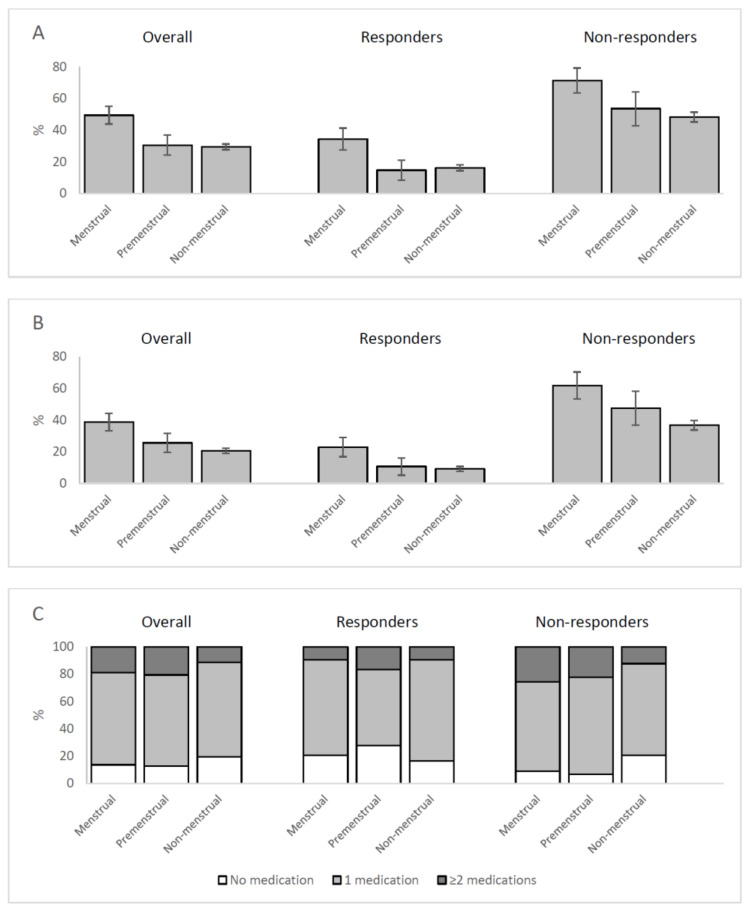
(**A**) Proportion of headache days in menstrual, premenstrual, and non-menstrual days in the study patients according to response to erenumab. (**B**) Proportion of moderate-to-severe headache days in menstrual, premenstrual, and non-menstrual days in the study patients according to response to erenumab. (**C**) Number of acute medications per headache day in menstrual, premenstrual, and non-menstrual days in the study patients, according to response to erenumab. All comparisons were significant (*p* < 0.001).

The proportion of headache days was higher in menstrual than in premenstrual or non-menstrual days (49.5% vs. 30.6% vs. 29.5%, respectively; *p* < 0.001). The finding was consistent in erenumab responders (34.4% vs. 14.8% vs. 16.3%, respectively; *p* < 0.001) and non-responders (71.4% vs. 53.6% vs. 48.3%, respectively; *p* < 0.001) (Figure 1A). Notably, the ORs showed that the difference was significant only between menstrual and non-menstrual days (Table 2).

The proportion of moderate-to-severe headache days was higher in menstrual than in premenstrual or non-menstrual days in the overall group (38.8% vs. 25.7% vs. 20.6%, respectively; *p* < 0.001), in erenumab responders (23.0% vs. 10.7% vs. 9.2%, respectively; *p* < 0.001) and in erenumab non-responders (61.9% vs. 47.6% vs. 36.8%, respectively; *p* < 0.001) (Figure 1B). Again, the ORs showed that the difference was significant only between menstrual and non-menstrual days (Table 2).

Figure 1C reports the number of medications used by patients per headache day. The proportion of headache days with ≥2 acute medications over total headache days was higher in menstrual or in premenstrual than in the non-menstrual days, in the overall group (19.0% vs. 20.6% vs. 11.4%; *p* = 0.009) and in erenumab non-responders (25.6% vs. 22.2% vs. 12.2%; *p* = 0.002), but not in erenumab responders (9.5% vs. 16.7% vs. 9.6%; *p* = 0.620). Compared to non-menstrual days, the difference was significant for both menstrual and premenstrual days in the overall group, for menstrual days only in erenumab non-responders, and for none of the categories in the erenumab responders (Table 2).

## 4. Discussion

Here, we present the first real-life data on the effect of erenumab on migraine, according to the different phases of the menstrual cycle. A subgroup analysis of a randomized controlled trial showed the efficacy of erenumab in menstrually-related migraine [19]. However, that study did not distinguish menstrual from non-menstrual days. Our data suggest that women with chronic migraine on treatment with erenumab had headaches more commonly in menstrual than in premenstrual or non-menstrual days. The pattern was similar in responders and non-responders to the treatment. Additionally, even in responders, moderate-to-severe attacks occurring on menstrual days were more common than those occurring on premenstrual or non-menstrual days. Notably, we observed a similar need for acute medication, and particularly repeated medication, in menstrual and in non-menstrual days in the subgroup of erenumab responders. This finding suggests that erenumab might improve the acute management of menstrual migraine in women who have a positive response to the treatment. This effect might be due to an overall reduced occurrence of attacks in responders and to a decreased attack duration or increased drug responsiveness during menstrual days, leading to less need for medication. This hypothesis is relevant as menstrual attacks are usually longer and less responsive to medication, as compared to non-menstrual attacks [12]. However, further studies comparing erenumab treatment with the pre-treatment status are needed to support this hypothesis.

Susceptibility to migraine pain is increased during the rapid estrogen decrease of the days preceding menstruation [11]. This increased susceptibility to migraine might depend on the increased CGRP levels. While some animal models suggested that the levels of CGRP are not influenced by estrogen levels throughout the menstrual cycle [20], several other models suggest that CGRP is upregulated when ovarian steroid levels fall [21,22,23,24,25,26]. Experimental evidence in humans suggests that trigeminovascular reactivity, an indirect measure of CGRP expression, peaks during the estrogen fall of the premenstrual period [27]; the fluctuations in trigeminovascular reactivity are particularly evident in menstrually-related migraine [28]. Taken together, evidence suggests that female sex hormones, and mostly estrogen, can modulate CGRP levels in women [29]. Hence, CGRP blockade might be counterbalanced by an increased expression of CGRP in the days around the menstruation. This might provide a biological explanation to the persistence of a menstrual pattern of migraine, despite treatment with a migraine-specific agent. Therefore, by hypothesis, hormonal treatments to prevent estrogen withdrawal [30] might be combined with erenumab to attain better migraine control.

Our study was limited by its small sample, which could not allow subgroup analyses according to the presence of aura, concomitant medication, or the potential benefit of erenumab dose escalation from 70 mg to 140 mg monthly [31]. Furthermore, dose escalation from 70 mg to 140 mg monthly was performed at variable time-points. We could also not directly compare erenumab responders with non-responders, but could only perform subgroup analyses, as the two groups were not matched. A further limitation of the study was the heterogeneous number of menstrual cycles contributed by each woman (Table 1). The small sample size implied that our findings should be confirmed in larger prospective studies, allowing those subgroup analyses and controlling for potential confounders. However, we considered the 2926 days of observation as statistical units, which improved the reliability of our findings. An additional study limitation, as previously stated, is that we could not compare data during treatment with erenumab with those preceding the treatment, as our patients did not provide information about their previous menstrual days. Moreover, we did not assess the difference between migraine and non-migraine headache; however, some patients find it difficult to distinguish between them. Additionally, our definition of ‘moderate-to-severe headache days’ likely included migraine days.

## 5. Conclusions

Our data showed that erenumab might improve menstrual migraine but that menstruation remains a trigger for migraine occurrence even after treatment with erenumab and even in those women who have a favorable response to the treatment.

## Figures and Tables

**Table 1:** Characteristics of the 18 included women.

No.	Age	Migraine Years	Medication Overuse	Aura	Concomitant Oral Preventives	Erenumab Dose (mg)	Responder	No. of Cycles
1	38	22	No	Yes	No	70	Yes	7
2	50	35	No	Yes	Yes	140	No	10
3	25	12	Yes	No	Yes	140	No	4
4	49	12	Yes	Yes	Yes	140	No	10
5	38	29	No	Yes	No	70	Yes	6
6	39	30	Yes	No	No	70	Yes	10
7	48	33	No	Yes	Yes	140	Yes	3
8	28	13	No	No	No	70	Yes	9
9	36	11	Yes	No	No	70	Yes	3
10	26	12	Yes	No	No	140	Yes	3
11	42	20	Yes	No	Yes	140	No	3
12	47	28	Yes	No	No	70	Yes	11
13	18	2	Yes	Yes	No	140	No	3
14	45	25	Yes	No	Yes	140	No	5
15	29	16	No	No	No	140	Yes	6
16	27	22	No	No	No	140	Yes	4
17	36	11	Yes	Yes	No	70	Yes	3
18	52	14	Yes	Yes	No	140	No	3

**Table 2:** Odds ratios and 95% confidence intervals for headache days and moderate-to-severe headache days, according to the phases of the menstrual cycle.

	Overall		Responders		Non-Responders	
	OR (95% CI)	*p* Value	OR (95% CI)	*p* Value	OR (95% CI)	*p* Value
*Headache days*						
Menstrual	2.34 (1.84–2.97)	<0.001	2.70 (1.93–3.78)	<0.001	2.67 (1.78–4.01)	<0.001
Premenstrual	1.05 (0.77–1.43)	0.751	0.89 (0.53–1.50)	0.663	1.23 (0.79–1.93)	0.358
Non-menstrual	1.00 (Ref.)	-	1.00 (Ref.)	-	1.00 (Ref.)	-
*Moderate-to-severe headache days*						
Menstrual	2.45 (1.91–3.14)	<0.001	2.94 (1.99–4.34)	<0.001	2.79 (1.90–4.09)	<0.001
Premenstrual	1.33 (0.96–1.85)	0.085	1.18 (0.65–2.15)	0.594	1.56 (1.00–2.44)	0.051
Non-menstrual	1.00 (Ref.)	-	1.00 (Ref.)	-	1.00 (Ref.)	-
*Headache days with ≥2 acute medications*						
Menstrual	1.82 (1.14–2.90)	0.012	1.00 (0.39–2.57)	0.992	2.46 (1.43–4.25)	0.001
Premenstrual	2.03 (1.06–3.89)	0.034	1.89 (0.51–7.04)	0.342	2.05 (0.96–4.35)	0.062
Non-menstrual	1.00 (Ref.)	-	1.00 (Ref.)	-	1.00 (Ref.)	-

## Data Availability

The data presented in this study are available on request from the corresponding author. The data are not publicly available due to privacy reasons.
